# Modeling the relationship between perceived service quality, tourist satisfaction, and tourists’ behavioral intentions amid COVID-19 pandemic: Evidence of yoga tourists’ perspectives

**DOI:** 10.3389/fpsyg.2022.1003650

**Published:** 2022-09-23

**Authors:** Ahmed Hassan Abdou, Shaimaa Abo Khanger Mohamed, Ayman Ahmed Farag Khalil, Azzam Ibrahem Albakhit, Ali Jukhayer Nader Alarjani

**Affiliations:** ^1^Department of Social Studies, College of Arts, King Faisal University, Al-Ahsa, Saudi Arabia; ^2^Department of Hotel Studies, Faculty of Tourism and Hotels, Mansoura University, Mansoura, Egypt; ^3^Department of Tourism Studies, Faculty of Tourism and Hotels, Mansoura University, Mansoura, Egypt

**Keywords:** yoga tourism, service quality, tourist satisfaction, behavioral intention, COVID-19, yoga retreats

## Abstract

**Purpose:**

This study aims to investigate the impact of perceived service quality (PSQ) on tourist satisfaction and behavioral intentions and explore the potential mediating role of tourist satisfaction in the relationship between service quality and behavioral intentions in the yoga tourism context during the COVID-19 pandemic. Further, this is to examine to what extent yoga tourist satisfaction directly affects their behavioral intentions.

**Design/methodology/approach:**

Based on a review of literature, the study proposes a conceptual model to test four hypothesized relationships among the constructs of perceived service quality, tourist satisfaction, and behavioral intentions. Data was collected by using a self-administrated questionnaire that was developed and directed to a convenience sample of yoga tourists (380 forms). Structural equation modeling (SEM) was employed to determine the relationship between study constructs.

**Findings:**

The results of SEM illustrated that all the hypothesized relationships are supported. The findings confirm that yoga tourists’ behavioral intentions are significantly affected directly and indirectly (through tourist satisfaction) by perceived service quality. Additionally, tourist satisfaction significantly partially mediates the relationship between PSQ and tourists’ behavioral intentions.

**Research limitations:**

The subject of this study was yoga tourists staying in yoga retreats/studios in Egyptian destinations (South Sinai Governorate). Future research may focus on other geographical destinations and other influential variables of yoga tourists’ satisfaction and behavioral intentions should be investigated.

**Practical implications:**

For improving tourists’ satisfaction and behavioral intentions, yoga service providers should take care by giving tourists personalized attention, and understanding, fulfilling their specific needs. Health and hygiene practices must be considered during the COVID-19 pandemic.

**Originality/value:**

This study is perhaps the first empirical study that examines the relationship between PSQ and tourists’ satisfaction and behavioral intentions in the yoga tourism context. A new integrated conceptual model that combined three service quality dimensions, namely, tangibles, intangibles as well as health and hygiene was developed and validated.

## Introduction

As a result of the multiplicity of tourists’ needs and desires, many types of tourism have emerged. One of these modern styles is yoga tourism. Yoga tourism is a unique type of tourism. In the past few years, yoga tourism has emerged as a new form of wellness tourism market that has grown rapidly (Jammu, 2016). Although yoga tourism has been frequently considered a niche within wellness tourism (Lehto et al., 2006; Smith and Kelly, 2006; Ali-Knight and Ensor, 2017), the yoga tourism and yogi tourists literature contexts had been included as a subset of several types of tourism such as holistic tourism (Kelly and Smith, 2009; Ali-Knight and Ensor, 2017), meditation tourism (Sharma and Nayak, 2019), spiritual tourism (Smith and Puczkó, 2009), medical tourism (Gautam and Bhatta, 2020), and special interest tourism (Ali-Knight, 2009).

Yoga is a science that has been practiced for more than 5,000 years (Lehto et al., 2006). Actually, it originated in India, and after that, it reached different nations all over the world (Jammu, 2016). The word ‘Yoga’ is derived from the Sanskrit root “Yuj,” which means “to unite” or “to join” (Dhiraj and Kumar, 2021). In other words, yoga means to unite the breath with the body, to unite the muscles with the mind, and most importantly to unite the self with God. Yoga is not a religion. It does not require a belief in a particular God or chanting particular mantras (Lehto et al., 2006). Widely, it was believed to provide a way for reducing stress, improving breath, and gaining flexibility (Lehto et al., 2006). Furthermore, it helps in healing from some health-related problems such as asthma, arthritis, lung inflammation, and chronic back pain (Ross and Thomas, 2010). Yoga has become the centerpiece of many people’s quests for a balanced life. Not only that, but yoga has also become an increasingly touristic phenomenon where people have not only practiced it daily but also on their vacations (Lehto et al., 2006).

Egypt is the land of Kemetic yoga. Kemetic yoga was developed by ancient Egyptians thousands of years ago (Siddiqui, 2021). Despite being practiced in fewer numbers than other yoga forms, Kemetic yoga has developed rapidly into the wonders of yoga tourism, which is now an integral part of wellness tourism (Komeil, 2021). Kemetic yoga is a regenerating and therapeutic yoga system derived from ancient Egyptians’ principles, philosophy, and science. It is distinguished by a series of geometrically progressive poses, breath guidance based on a four-part rule “We Inhale; Hold the Breath, Exhale; Hold the Breath,” and tongue connection (Barga, 2021). In addition to Kemetic yoga, there are many types of yoga practiced in Egyptian tourist destinations (i.e., Hatha, Iyengar, Vinyasa, Nidra…etc.).

During the COVID-19 pandemic, the increasing numbers of COVID-19 cases and deaths besides mandatory social distancing measures have led to rising stress, anxiety, loneliness, and depressive feelings (Shinn and Viron, 2020). Several measures for physical and mental well-being are described, including pranayama and meditation (Tillu et al., 2020). Pranayama **”***the practice of breath regulation and the main component of yoga***”** is known to improve lung functions and strengths muscles of respiration (Santaella et al., 2011, p. 2). In the meditation context, different studies confirmed that meditation had reduced anxiety, depression, stress, cortisol level as well as blood pressure (Pascoe et al., 2017; Behan, 2020). The practice of yoga, including meditation, could serve as a simple and useful home-based solution for preventing and managing post-recovery stress of COVID-19 (Tillu et al., 2020). Pandemic-related closures and restrictions on face-to-face activities have led yoga centers to stop their business, either temporarily or permanently (B2B News, 2022). In response, yoga businesses have moved online as a safer alternative to face-to-face classes. During the pandemic, reservations for yoga online classes have grown by 25% as more people joined from their homes (B2B News, 2022). Virtual yoga classes become more popular now than they were before the pandemic, and this trend is likely to continue post-pandemic (B2B News, 2022).

Recently, service quality has received considerable attention from researchers and professionals in the extremely competitive marketplace. By focusing on the provision of superior service quality, a company can achieve a sustained competitive advantage, distinguish itself from the competitors, and improve efficiency (Kandampully and Suhartanto, 2000). A highly perceived service quality (PSQ) will result in improved customer satisfaction and retention, larger market share, positive word-of-mouth (WoM), decreased employee turnover, reduced operational expenses, and consequently improved financial performance and profitability (Getty and Thompson, 1994; Duncan and Elliott, 2002; Albacete-Saez et al., 2007).

Based on reviewing the tourism literature, numerous scholars have examined the causal relationship between service quality, tourist satisfaction, and behavioral intentions in different tourism contexts. In the medical tourism context, Han and Hyun (2015) developed a model to examine the impact of PSQ and medical travelers’ satisfaction on their intentions to revisit the destination country. They concluded that PSQ and satisfaction significantly affect travelers’ intentions to revisit. Furthermore, satisfaction acted as a significant mediator between PSQ and travelers’ behavioral intentions. In Malaysia’s rural tourism, the empirical study conducted by Osman and Sentosa (2013) confirmed that tourist satisfaction has a significant and positive partial mediator of both service quality and tourist loyalty relationships. In the context of sports tourism, Jeong et al. (2019) concluded that event quality and tourist satisfaction significantly positively affect tourists ‘behavioral intentions. Further, the findings of structural equation modeling (SEM) showed the partial mediating role of tourist satisfaction in the relationship between event quality and behavioral intentions. In health and wellness tourism, Quintela et al. (2010) indicated that higher PSQ is one of the main predictors of tourist satisfaction and behavioral intentions. In the Edu-tourism context, a study was conducted to investigate the relationship between the university’s service quality, Edu-tourist satisfaction, recommendation, and repurchase intentions illustrated that university service quality positively influences Edu-tourist satisfaction and behavioral intentions (Rahimizhian et al., 2020). In addition, Edu-tourist satisfaction positively influences recommendation and repurchase intentions. Moreover, Edu-tourist satisfaction significantly mediated the impact of service quality on recommendation and repurchase intentions. In the context of cultural heritage tourism in Indonesia, Canny (2012) revealed that local tourists’ satisfaction, as well as their future behavioral intentions (i.e., do recommend, say positive WOM, and revisit tourist destination), are significantly affected by PSQ.

Although numerous studies have examined the relationship between service quality, tourist satisfaction, and behavioral intentions in different tourism contexts, there are still gaps in tourism academic literature to understand the interrelationships between these variables in the yoga tourism context, particularly in the era of COVID-19. Furthermore, the relationship between the three constructs is still in debate. Although numerous scholars illustrated the positive relationship between PSQ and customer satisfaction, some studies asserted that the two constructs are likely to be positively correlated, but unlikely to be linear (Crompton and Love, 1995). Regarding the relationship between customer satisfaction and behavioral intentions, some researchers found a direct positive relationship, others found a negative relationship (where dissatisfied customers engage in more WoM), and other studies have not confirmed the significant direct relationship between them (i.e., Sivadas and Baker−Prewitt, 2000). Additionally, in the previous studies investigating tourist satisfaction as a mediator between PSQ and behavioral intentions, mixed conclusions have been reached, consequently, further research is needed. Accordingly, to fill this gap in the tourism academic literature, the current study aims to investigate the impact of PSQ on tourist satisfaction and behavioral intentions and explore the potential mediating role of tourist satisfaction in the relationship between PSQ and behavioral intentions in the yoga tourism context during the COVID-19 pandemic, in a sample of Egyptian tourist destinations, particularly, South Sinai governorate. Further, this is to examine to what extent yoga tourists’ satisfaction directly affects their behavioral intentions. To achieve this aim, a self-administrated questionnaire will be used for data collection from a convenience sample of yoga tourists, and the SEM will be employed to examine the relationship between the study constructs.

This study effectively contributes to the literature on yoga tourism in various ways. Firstly, to the best of our knowledge, this is the first study that examines the direct and indirect effect of PSQ on yoga tourists’ behavioral intentions, particularly in the era of the COVID-19 pandemic. Secondly, the study introduces a new integrated conceptual model that combined three service quality dimensions, namely, tangibles, intangibles as well as health and hygiene that could help tourism scholars as a basis for further studies aiming to examine the justifications for improving tourists’ satisfaction and behavioral intentions in yoga and various tourism contexts. Thirdly, the findings of the study could help yoga service providers in exploring the factors influencing yoga tourists’ satisfaction and behavioral intentions.

The study is divided into six sections. Section “Introduction” briefly summarizes the introduction. In section “Theoretical background and hypothesis development,” we discuss the theoretical background related to the concept of yoga tourism, and the relationship between service quality, tourists’ satisfaction, and behavioral intentions in the tourism industry. In Section “Materials and methods,” the measures, sampling, and data collection methods are described. Furthermore, the study’s results are summarized and analyzed in Section “Results.” Discussions of the study’s results and theoretical as well as practical implications are presented in Section “Discussion and implications.” Section “Limitations of the study and further research” focuses on the limitations of the study and further research.

## Theoretical background and hypothesis development

### The concept of yoga tourism

In the past few years, yoga tourism has emerged as a new form of wellness tourism market that has grown rapidly (Jammu, 2016). Smith and Kelly (2006, p. 17) defined yoga tourism as **”***tourism which focuses on the union of body, mind, and spirit, but which is essentially areligious*.” However, Ali-Knight (2009, p. 87) defined it as **”***travel to a destination to engage in the practice of yoga and in related activities that will enhance the physical, mental or spiritual wellbeing of the tourist.”* Jammu (2016) described yoga tourism as an act in which people travel to other countries for spiritual and medical treatments with the help of yoga, as well as to tour, vacation, and experience the attractions of the countries in which they are traveling.

Regarding the benefits of yoga tourism, Smith and Kelly (2006) illustrated that yoga tourism is a self-guided journey with transformative capabilities on all levels; physically, mentally, spiritually, and socially. This transformation has been built on the concept that yoga is a practice that can change gradually the life of human beings and societies as well. This transformation may begin with a series of postures for the physical body, but if it is regularly practiced, it can lead to mental and spiritual transformation as well (Smith and Sziva, 2016). Even medical science is initially beginning to recognize the benefits of yoga for physical and mental well-being, e.g., for back pain, stress, depression, anxiety, and hypertension (Taneja, 2014).

Concerning the characteristics of yoga tourism, the findings of the empirical study carried out by Ali-Knight and Ensor (2017) indicated that most of the investigated participants agreed and strongly agreed that they seek their travel new experiences and destinations, to have the freedom to do their things, interacting with local people and learning about their traditions and cultures, accommodating all-inclusively in yoga retreats, and avoiding worry about anything through traveling in an organized trip. The financial considerations, time, and accommodating partners/children were the main challenges faced by their engagement in yoga tourism. Furthermore, the qualified and experienced teacher, healthy food, and a safe destination were respectively the highest important factors to choose a yoga trip.

### Perceived service quality

In recent years, researchers and professionals have paid considerable attention to service quality. The concept of service quality has been defined in different ways. (Drucker (1985), p. 228) stated that **”***quality in a product or service is not what the supplier puts in. It is what the customer gets out and is willing to pay for*.” Grönroos (1984, p. 37) defined PSQ as **”***the outcome of an evaluation process, where the consumer compares his expectations with the service he perceives he has received, i.e., he puts the perceived service against the expected service. The result of this process will be the perceived quality of the service*.” Moreover, it was frequently cited as the difference between customers’ expectations and perceptions of provided service (Parasuraman et al., 1985). This description had been utilized in different research as a measurement tool for service quality. Based on this conceptualization, a service quality measurement scale (SEVQUAL) including 22 items classified into five dimensions namely, reliability, assurance, tangibility, responsiveness, and empathy, had been developed (Parasuraman et al., 1988). Although it the widely used of this scale to measure service quality, a quite number of criticisms have been met because of its validity and reliability (Cronin and Taylor, 1992; Jain and Gupta, 2004). Furthermore, Buttle (1996) illustrated that the SERVQUAL scale does not appear to be universal because of service quality dimensionality which differs according to the kind of service examined.

Several scholars have attempted to determine the dimensions of service quality that customers consider when they assess the quality-of-service experience. Grönroos (1984) and Zaibaf et al. (2013) categorized service quality into three dimensions as follows; technical quality **”**the quality of what consumers receive as a result of their interaction,” functional quality **”**how a customer gets the technical outcome functionally,” and corporate image **”**refers to the outcome of how the consumer perceives the company.” Parasuraman et al. (1988) classified it into five distinct dimensions namely, tangibles **”**physical facilities, equipment, and appearance of personnel**,”** reliability **”**ability to perform the promised service dependably and accurately,” responsiveness **”**willingness to help customers and provide prompt service,” assurance **”**knowledge and courtesy of employees and their ability to inspire trust and confidence**”** and empathy **”**caring, as well as the individualized attention the company provides its customers.” Marić et al. (2016), in their empirical study, developed a measurement model composed of two dimensions namely, tangibility and in-tangibility. The term **”**tangibility**”** generally refers to aspects of services, such as the appearance of the staff, tools and equipment, ventilation, lighting, and other physical elements used to provide the services. Intangibility reflects the interrelationship between staff and customers which include handling customer complaints properly, providing all required information, paying adequate attention, understanding the specific needs of the customers….etc.

### Tourist satisfaction

As a psychological concept, satisfaction is described as the pleasure and feeling of well-being that one experiences from receiving what he/she expects from an appealing product or service (Chi and Qu, 2008). Ardani et al. (2019) revealed that customers’ satisfaction is derived from comparing their expectations before and after consumption. Dissatisfied customers mean that customers have been got less than they expected. In other words, when they find that product and service performance doesn’t match and meet their expectations, they will feel dissatisfied. Meanwhile, satisfied customers refer to customers who got only what they expected. If product and service performance exceed customers’ expectations, they will be more delighted and highly satisfied (Kopalle and Lehmann, 2006). Gundersen et al. (1996, p. 74) defined consumer satisfaction as **”***a post-consumption evaluative judgment concerning a specific product or service*.” Moreover, customer satisfaction refers to the subjective and emotional state of consumers toward their needs and wants (Giese and Cote, 2000).

In the tourism industry, satisfaction is a highly sensitive issue where it was recognized as a driving force in tourists’ loyalty (Adinegara et al., 2021). Tourist satisfaction has a significant impact on tourists’ choice of destinations, as well as their decision to recommend them to their close friends and acquaintances (Amirreza et al., 2013; Han and Hyun, 2015). The antecedent factors affecting tourists’ satisfaction are still subject to debate. A study conducted by Aliman et al. (2014) aimed to explore the impacts of the image of the destination, tourist expectations, and service quality perceived, on the satisfaction of tourists and illustrated that all these variables had a positive and significant relationship with tourist satisfaction.

### Tourists’ behavioral intentions

Based on the literature review, tourists’ behavioral intentions refer to what they intended to do after having an experience (Naik et al., 2010). To fully understand tourist behavior or motivation in the future, the behavioral intention must be analyzed (Afshardoost and Eshaghi, 2020). The ability to properly understand and analyze tourists’ behavioral intentions is the key to determining the development of the tourism industry and tourists’ satisfaction (Jeong et al., 2019). As mentioned by Zeithaml et al. (1996), future behavioral intentions indicate that the customers’ loyalty will drive them to give recommendations to others. As a result, it will positively influence people, inspire friends and families to spend more money, and ultimately impacts the future consumption of products or services.

Numerous studies have measured the tourists’ behavioral intention through three variables as follows; (1) the tourist intention to visit **”***is a tourist behavioral intention that exists before a tourist takes a trip and is influenced by tourist destination image*,” (2) the intention to revisit **”***tourists repeat their actions where they want to revisit a tourist destination that has been visited before*,” and (3) intention to recommend **”***the intention of tourist to recommend the destination to their friends and families when they have a positive perception toward their experience***”** (Wang and Hsu, 2010; Chen et al., 2014; Dolnicar et al., 2015; Hsu et al., 2016; Stylos et al., 2016). In addition, Jalilvand et al. (2012) stated that positive WoM regarding a tourist destination is also an example of a behavioral intention.

According to Chen and Chen (2010), behavioral intentions could be classified into favorable and unfavorable. Favorable behavioral intention is demonstrated by positive WoM, spending more money on the company’s products and services, paying a premium price, and staying loyal to the company’s brand. On the other hand, spending less money, leaving the company, spreading negative WoM, and filing lawsuits are all examples of unfavorable behavioral intentions (Ladhari, 2009). In the highly competitive marketplace, to survive and grow, companies should maintain and retain their loyal customers (Velázquez et al., 2011).

### The relationship between perceived service quality and tourist satisfaction

Within the context of the tourism and hospitality industry, it could be noticed that different studies have examined the causal relationship between PSQ and customer satisfaction. In earlier studies, PSQ has been identified as one of the key factors for improving tourist satisfaction (i.e., González et al., 2007; Zaibaf et al., 2013). A study conducted on a convenience sample including 456 tourists from China concluded that PSQ of public health services had a significant and positive link with tourists’ satisfaction (*B* = 0.341, p < 0.05) (Han et al., 2021). The higher level of perceived public health services quality increases tourists’ satisfaction. In the health and wellness tourism context, items related to the willingness of employees to provide service promptly, the ability of employees to perform dependably and accurately, and providing individualized tourist attention represent the highest predictors of satisfaction among Portuguese tourists (Quintela et al., 2010). Furthermore, the results of the empirical study aimed at identifying the impact of PSQ on tourist satisfaction and retention in the Maldives tourism industry confirmed that three service quality dimensions, namely responsiveness, reliability, and tangibility significantly and positively affect tourist satisfaction (Ibrahim et al., 2015). However, assurance and empathy have no impact on tourist satisfaction. In Malaysian rural tourism, the findings of Osman and Sentosa’s study (2013) illustrated that PSQ has a strong positive and significant direct impact on tourists’ satisfaction (*B* = 0.787, *P* < 0.000). The study suggests that for increasing and enhancing rural tourists’ satisfaction, factors of service quality should be improved and considered. In the restaurant context, a study carried out on 309 customers aimed to identify the impact of PSQ on customer satisfaction illustrated that PSQ (i.e., food quality, employee service quality, timeliness…. etc.) significantly and positively affects customer satisfaction (Tuncer et al., 2021). Khuong and Phuong (2017) in their study on 1673 tourists in Vietnam indicated that PSQ significantly and positively correlated to tourist satisfaction. Sadeh et al. (2012), in their study aimed to determine the factors affecting Iranian tourists’ satisfaction, revealed that PSQ is one of the main predictors of tourist satisfaction. They assured that if tourists don’t feel that the quality received exceeds the money paid, satisfaction will not be achieved. Accordingly, it seems reasonable to suggest that.

*H_1_: Perceived service quality significantly and positively affects yoga tourists’ satisfaction.*

### The relationship between perceived service quality and tourists’ behavioral intentions

Numerous scholars explored and investigated the relationship between PSQ and behavioral intentions and conclude the significant direct, and indirect effects of service quality dimensions on customers’ loyalty, purchase and repurchase intentions, willingness to pay more money, recommendations to others, and positive WoM (i.e., Zeithaml et al., 1996; Baker and Crompton, 2000; Bei and Chiao, 2001; Alexandris et al., 2002; Ladhari, 2009). Findings of the empirical study carried out by Alexandris et al. (2002) in the Greek hotel industry concluded that SERVQUAL dimensions positively and significantly affect tourists’ behavioral intentions specifically WoM communication and purchasing intent. Furthermore, a study conducted by Ladhari (2009) on a sample of Canadian travelers confirmed that service quality directly and indirectly (through emotional satisfaction) affects travelers’ behavioral intentions. Meeprom and Silanoi (2020) investigated the perceived quality of a special event and its influence on behavioral intentions in Thailand and concluded that perceived quality significantly and directly impacts tourists’ behavioral intentions. In Spain, González et al. (2007) empirically assessed tourists’ behavioral intentions through PSQ and customer satisfaction in a sample of Spanish spa resorts. The findings of the study revealed that PSQ positively and significantly influences WoM communication, buying intentions, and price sensitivity as predictors of behavioral intentions, respectively. With regards to this, we propose the following hypothesis:

*H_2_: Perceived service quality significantly and positively affects yoga tourists’ behavioral intentions.*

### The relationship between tourist satisfaction and behavioral intentions

Considering its importance in gaining market share in tourism, practitioners and academics have paid considerable attention to the relationship between tourist satisfaction and behavioral intentions (Ardani et al., 2019). Tourist satisfaction played an important role in the sustainability of tourists’ loyalty (Baker and Crompton, 2000). Olorunniwo et al. (2006) demonstrated that although service quality significantly affects behavioral intentions, the indirect impact (through satisfaction as a mediator) was a stronger predictor for the behavioral intents in the service factory context. The level of customer loyalty is determined by the consumer’s preferences, and the degree of satisfaction with the product or service (Meeprom and Silanoi, 2020). As a result, loyalty is a critical outcome of satisfaction which is manifested through repeat purchases, willingness to pay premium prices, and readiness to recommend the destination to others (Osman and Sentosa, 2013). Moreover, an empirical study carried out by Viana et al. (2021) demonstrated that tourist satisfaction had positive and significant impact on tourist behavioral intention. Studies revealed that satisfaction is a key predictor of behavioral intention which enhances the intention to revisit and recommend to other tourists by using positive WOM advertising. A tourist will revisit when it satisfies with his/her visit (Tian-Cole et al., 2002; Cole and Illum, 2006; Osman and Sentosa, 2013). Upon the previous findings, we hypothesize that:

*H_3_: Yoga tourists’ satisfaction significantly and positively affects tourists’ behavioral intentions.*

### The mediating role of tourist satisfaction in the relationship between perceived service quality and behavioral intentions

In the tourism context, various scholars indicated that satisfaction plays a mediating role in the relationship between PSQ and behavioral intentions. Cronin et al. (2000) suggested that the indirect impacts of service quality (through customers’ satisfaction) enhanced their effects on behavioral intentions. Tian-Cole et al. (2002) empirically investigated the interrelationship between service quality, satisfaction, and behavioral intents among 282 tourists in Wildlife Refuge. They concluded that PSQ had direct and indirect (through satisfaction) significant and positive impacts on tourists’ behavioral intentions. Among forest visitors (*n* = 395), Lee et al. (2004) found that tourist satisfaction partially plays a mediating role between service quality and behavioral intentions. Similar results were also found in Alén’s (2018) study which concluded that the relationship between PSQ and tourists’ behavioral intentions is partially mediated by tourist satisfaction. The same results have been observed in the context of sports tourism where Jeong et al. (2019) concluded that tourist satisfaction partially mediates the link between behavioral intents and the quality of events. However, Cole and Illum (2006) stated that the impact of service quality on behavioral intentions (positive WOM and intentions to revisit) was fully mediated by the satisfaction of festival visitors. Hence, we imply the following hypothesis:

*H_4_: Tourist satisfaction significantly mediates the positive relationship between perceived service quality and yoga tourists’ behavioral intentions.*

**FIGURE 1:**
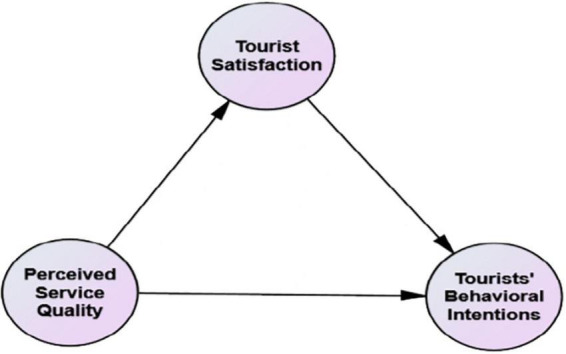
The research conceptual model.

The conceptual model of research is presented in Figure 1.

## Materials and methods

### Measures and instrument development

The data was collected in this study by using a self-administrated questionnaire. The structure of the questionnaire form was developed by extensively reviewing the study literature to identify valid and frequently used measures. The questionnaire form was composed of five sections. The first section dealt with the participants’ demographic data which included gender, age, level of education, nationality, and marital status. The second section sought to identify the main motivation to participate in yoga trips and the vital factors that affect their choice of yoga trips. The third section aimed to explore the PSQ. The fourth and fifth sections focused on identifying tourists’ satisfaction and behavioral intentions respectively.

In response to the COVID-19 pandemic, the PSQ scale (SERVPERF) developed by Cronin and Taylor (1992) in addition to some measures related to health and hygiene focused on preventing and mitigating COVID-19 in the workplace in accordance with World Health Organization [WHO] (2021) were adapted and used to explore the perceptions of the investigated participants toward variables of service quality. The initial scale was composed of (28) items including (22 items of the SERVPER scale plus six items concerning health service quality. To determine the items constituted in yoga tourism, exploratory factor analysis (EFA) was employed with a principal component and Varimax rotation. As a result, the scale has been reduced to (16) items categorized into three dimensions namely, tangibles composed of four items (i.e., **”**the physical facilities and accessories in a yoga retreat/studio are visually appealing**”**), in-tangibles composed of nine items (i.e., **”**staff shows a sincere interest in solving your complaints**”**), and health and hygiene included three items **”**i.e., protective masks and hand sanitizers are usually available for all guests and staff**”**). The internal consistency reliability (Cronbach’s alpha) for the PSQ scale was 0.925.

In accordance with Wang and Hsu (2010) and Nguyen et al. (2021), an adapted three-item measure scale was utilized to identify tourists’ satisfaction as follows; **”**The yoga retreat/studio and its staff had met all our expectations,” **”**compared with other retreats, the level of satisfaction was amazing and overall,” **”**I am satisfied with my experience during this travel.” The internal consistency reliability (Cronbach’s alpha) for the tourists’ satisfaction scale was 0.883.

Tourists’ behavioral intentions were assessed by using an adapted three-item measure scale based on Papadimitriou et al. (2015) and Sharma and Nayak (2019). These items reflect the intention to revisit**”** If had to decide again I would revisit this retreat,” the recommendation to others **”**I will certainly recommend this retreat to my friends and acquaintances,” and positive WoM communication **”**I will speak positively of the retreat to friends and relatives.” The internal consistency reliability (Cronbach’s alpha) for tourists’ behavioral intentions scale was 0.782. All of the study scales’ items have been measured by using a five-point Likert scale ranging from 1 = strongly disagree, to 5 = strongly agree was used.

The survey instrument was originally prepared in English and then translated into Italian, Russian, and Deutsch languages (where the majority of tourists come) and then reverse translated from previous languages to English to confirm that there were no differences in meaning. The content validity of the questionnaire form was examined to ensure clarity and suitability of the questionnaire. Six tourism experts and thirty-five tourists pre-tested the instrument. They asked to assess the content validity of the questionnaire form and provide any feedback. Upon the participants’ comments, the wording of some statements was modified, and some statements were re-ordered.

### Sampling and data collecting

As mentioned previously, the current study aims to investigate the impact of PSQ on tourist satisfaction and behavioral intentions and explore the potential mediating role of tourist satisfaction in the relationship between service quality and behavioral intentions in the yoga tourism context during the COVID-19 pandemic, in a sample of Egyptian tourist destinations, particularly, South Sinai governorate.

As published on the State Information System (2022), South Sinai is a global hub for all kinds of tourism, hosting all climatic, natural, cultural, and marine attractions. It is located in the southern half of the Sinai Peninsula. In addition to beautiful beaches, mountains, plains, and valleys, the region boasts the water of the Red Sea, where coral reefs and rare fish can be found. The most important tourist destinations in the governorate are concentrated in the Golden Triangle including, Sharm El Sheikh, Nuweiba, and Dahab, where most of the yoga retreats are located. The region is among the fastest-growing tourist destinations worldwide, with about 90% of Egyptian tourism investment concentrated in the coastal resorts of South Sinai (Shackley, 1999). Before the COVID-19 pandemic, visitor arrivals to Egypt recorded 1,3026 million tourists in December 2019. The largest source of tourists in South Sinai is Western Europe, followed by Eastern Europe, and the United States, respectively (Elnagar and Derbali, 2020).

To achieve the aim of the study, a self-administrated questionnaire was developed and directed to a sample of yoga retreat guests. The convenience sampling technique was used. Through the relationships of the research team with yoga service providers, they were asked to permit to distribute the questionnaire among yoga retreat guests during the check-out process.

The appropriate sample size according to the recommendation of Hair et al. (2013) was decided. They recommended calculating the appropriate sample size based on the number of the investigated variables. The minimum ratio (variable: sample = 1:10) is acceptable. Consequently, the minimum sample size required for this study was 220 participants, where the total variables under investigation are 22 variables.

Participants were told that participation in the study is voluntary. A total of 380 questionnaires were distributed, only 317 forms were valid for statistical analysis with a response rate of 83.4%. The investigated participants were assured that all collected data would remain anonymous, confidential, and used only for research purposes, to reduce the risk of common method variance/bias (CMV). They were asked to answer the questions honestly, and there is neither correct nor incorrect answers. Furthermore, Harman’s single-factor test as a widespread and simple statistical tool that discovers CMV was utilized (Rodríguez-Ardura and Meseguer-Artola, 2020). Data collection spanned almost 3 months (November 2021–January 2022).

The sample of this study was composed of 317 participants. Regarding their gender, the majority of the investigated respondents (*N* = 229, 72.2%) were females and 27.8% (*N* = 88) were males. In terms of their ages, yoga practitioners having an average age range from 30 to 40 years represent the higher category (*N* = 171, 53.9%). In the context of the level of education, the vast majority (*N* = 258, 81.4%) had a college education. Concerning their marital status, 67.5% (*N* = 214) were single. In terms of nationality, most of them come from Eastern Europe (55.2%, *N* = 175).

### Data analysis

The collected data were analyzed by using SPSS v. 20 and Amos v. 24. To represent the sociodemographic data of the research participants, identify the main motivation to participate in yoga trips, determine the main factor that affects their choice of yoga trips as well as explore tourists’ perceptions toward study constructs, descriptive statistics, including mean, standard deviation were utilized. The EFA was used to explore the fundamental dimensions of PSQ. The validity and reliability of measurement items were confirmed by confirmatory factor analysis (CFA) and reliability analysis (Cronbach’s alpha). CMV was examined by Harman’s single factor test. Composite reliability (CR) and average variance extracted (AVE), were calculated for validity confirmation. Discriminant validity based on Fornell–Larcker criterion was also examined. Finally, to determine the direction as well as the relationships between study constructs SEM was employed.

## Results

### Exploratory factor analysis

To explore the fundamental dimensions of PSQ, the EFA was employed using SPSS 22.0. Firstly, to determine the suitability of the respondent data for factor analysis, the Kaiser–Meyer–Olkin (KMO) Measure of Sampling Adequacy has been examined. As recommended by Williams et al. (2010), the results presented in Table 1 demonstrated the suitability of data for factor analysis (KMO = 0.900; *P* < 0.001).

**TABLE 1:** Kaiser–Meyer–Olkin (KMO) and Bartlett’s test.

Kaiser–Meyer–Olkin measure of sampling adequacy		0.900
**Bartlett’s test of sphericity**	Approx. Chi-Square	5726.157
	Df	120
	Sig.	0.000

In principal components factor analysis with a varimax rotation, three factors, with cut-off factor loadings of 0.50 and eigenvalues greater than one, explained 78.74% of the variance of PSQ. As shown in Table 2 the Cronbach’s alpha was above the threshold value of 0.70 (Tangibles = 0.928, Intangibles = 0.957, and Health and Hygiene = 0.892), assuring the high reliability of the results (Hair et al., 2013). Table 2 presents the results of EFA for three factors, namely tangibles and intangibles, and health and hygiene.

**TABLE 2:** Exploratory factor analysis (EFA) results of perceived service quality.

Factor	Items	Factor loading	% of variance explained	Eigenvalue	Cronbach’s alpha
(1) Tangibles	Tang1	0.814	21.048%	6.727	0.928
	Tang2	0.858			
	Tang3	0.885			
	Tang4	0.917			
(2) Intangibles	In-tang1	0.853	42.046%	3.368	0.957
	In-tang2	0.845			
	In-tang3	0.865			
	In-tang4	0.937			
	In-tang5	0.935			
	In-tang6	0.928			
	In-tang7	0.763			
	In-tang8	0.769			
	In-tang9	0.799			
(3) Health and Hygiene	Health1	0.854	15.644%	2.503	0.892
	Health2	0.896			
	Health3	0.834			

Total explained variance = 78.738%.

### Motivators for participating in yoga trips

This question aimed to determine the main reason which motivated the investigated respondents to participate in yoga trips. The participants were asked to only select one reason. The four motivation factors which had emerged from the analysis conducted by Lehto et al. (2006) namely, **”**seeking spirituality,” **”**enhancing mental well-being,” **”**enhancing physical condition,” and **”**controlling negative emotions**”** had been addressed. As shown in Table 3, the majority of the investigated respondents illustrated that enhancing physical condition was the most motivator for participating in yoga trips, constituting 47.3% (*N* = 150). Enhancing mental well-being, controlling negative emotions as well as seeking spirituality represented 32.5, 12, and 8.2% respectively.

**TABLE 3:** Motivators for participating in yoga trips.

Factor	Frequency	%
Enhancing physical condition	150	47.3
Enhancing mental well-being	103	32.5
Controlling negative emotions	38	12
Seeking spirituality	26	8.2
Total	317	100

### Factors affecting the choice of yoga trips

This question was developed to explore the vital factors that affect the investigated respondent during their choice of yoga trips throughout the COVID-19 pandemic. Some factors had been addressed by Ali-Knight and Ensor (2017) plus some of the preventative measures had been adapted and considered. The findings of the investigated respondents have been shown in Table 4.

**TABLE 4:** Factors affecting the choice of yoga trips.

Factor	Mean	Standard deviation
Health and hygiene regulations (COVID-19 preventative measures)	4.45	0.812
Climate and the natural attractions in yoga destination	4.39	0.778
Professional and well-experienced teachers	4.37	0.815
Safety in a tourist destination	4.34	0.837
Health and quality of food provided	4.29	0.884
Accommodation services and facilities included	4.25	0.886
Recreational activities and cultural attractions	4.01	1.04
The popularity of yoga destination	3.98	0.975
Yoga styles offered	3.96	0.910
Secluded and unspoiled retreat/studio	3.94	0.834
Spa and alternative health treatment	3.74	0.840
Accessibility to yoga destination	3.65	1.25

Responses to the COVID-19 pandemic, the results presented in Table 4, illustrated that the majority of the investigated respondents strongly agreed (*M* = 4.22), that health and hygiene regulations (COVID-19 preventative measures) adopted have become the most important factor influencing their choice of yoga trips. Furthermore, they strongly agreed that climate and the natural attractions in yoga destinations, the professionalism of the yoga teachers, safety in the tourist destinations, health and quality of food provided, as well as the accommodation services and facilities included representing the highest important factors that influence their decision when choosing the yoga trip respectively.

### Descriptive statistics

Table 5 shows the mean and standard deviation of all variables related to the study’s constructs. Regarding the PSQ, the majority of the investigated respondents agreed and strongly agreed on items related to the tangibles dimension where the average mean ranged from 4.24 to 4.15. The highest item ranked linked to tangibles dimension was **”**a yoga retreat has comfortable furniture, fixture, and appropriate atmosphere**”** with an average mean of 4.24, followed by **”**staff was well dressed and have a neat professional appearance,” and **”**the physical facilities and accessories in a yoga retreat were visually appealing**”** with an average mean 4.22 and 4.21 respectively. Moreover, they agreed on all items related to an intangibles dimension where the average mean ranged from 3.83 to 4.13. They ranked **”**staff shows a sincere interest in solving guests’ complaints**”** as the highest attribute (*M* = 4.13). Higher PSQ has been considered to be health and hygiene service quality attributes. The investigated respondents strongly agreed on all of the items with an average mean has been raised to 4.35. The yoga service providers were keen to keep rooms, bathrooms, and public areas usually cleaned and well sanitized. Overall, a good level of PSQ has been achieved (*M* = 4.09, *SD* = 0.716).

**TABLE 5:** Descriptive statistics, reliability, and confirmatory factor analysis properties.

Constructs	Items	*M* ^1^	*SD* ^2^	Std. Loading (CFA)^3^	*t*-value	Cronbach’s alpha	CR^4^	AVE^5^
***Perceived Service Quality*** *(M* = *4.09, SD* = *0.716, Cronbach’s alpha* = *0.925, CR* = *0.977, AVE* = *0.726)*								
Tangibles	The physical facilities and accessories in a yoga retreat were visually appealing	4.21	0.94	0.782	F	0.928	0.932	0.775
	The appearance of the physical facilities was in keeping with the type of services provided	4.15	0.94	0.839	16.844***			
	A yoga retreat had comfortable furniture, fixture, and an appropriate atmosphere	4.24	0.90	0.934	19.5***			
	The staff was well dressed and have a neat professional appearance	4.22	0.88	0.955	20.037***			
Intangibles	When the staff promises to do something by a certain time, it does so	4.04	1.14	0.852	F	0.957	0.954	0.701
	The staff shows a sincere interest in solving guests’ complaints	4.13	1.11	0.777	17.434***			
	The staff provides services at the time they promise to do so	4.07	1.09	0.802	18.366***			
	The staff never be too busy to respond to your requests	3.86	1.16	0.992	28.209***			
	The staff was able to understand your specific needs.	3.84	1.16	0.988	27.96***			
	The yoga retreat had a staff who give you a personal attention	3.83	1.18	0.975	27.042***			
	The staff has the skills and adequate knowledge to perform the service professionally	3.93	1.21	0.647	13.339***			
	The behavior of staff instilled confidence among guests	3.93	1.28	0.662	13.754***			
	The staff tells you exactly the right schedule of the services to be performed	4.02	1.09	0.751	16.527***			
Health and hygiene	Protective masks and hand sanitizers were available for all guests and staff	4.31	0.80	0.795	F	0.892	0.894	0.738
	The retreat space and seating capacity are limited to allow for social distancing	4.32	0.83	0.895	17.504***			
	Rooms, bathrooms, and public areas were usually cleaned and well sanitized	4.35	0.82	0.883	17.324***			
Tourist satisfaction	The yoga retreat/studio and its staff have met all our expectation	4.01	0.93	0.933	F	0.883	0.888	0.727
	Compared with other retreats, the level of satisfaction in this place was amazing	4.08	0.98	0.754	16.54***			
	Overall, I am satisfied with my experience during this travel	4.02	0.93	0.861	20.189***			
Tourists’ behavioral Intentions	If had the opportunity to decide again I would revisit this retreat/studio	4.20	0.87	0.746	F	0.782	0.786	0.552
	I will certainly recommend this retreat/studio to my friends and acquaintances	4.24	0.88	0.776	12.484***			
	I will speak positively of this retreat/studio to friends and relatives	4.17	0.91	0.704	11.47***			

M^1^, mean; SD^2^, standard deviation, Std. Loading; (CFA)^3^, standardized factor loading; CR^4^, composite reliability; AVE^5^, average variance extracted. x^2^ = 486.312 (df = 201) p < 0.001, x^2^/df = 2.419, Comparative Fit Index (CFI) = 0.958, Normed Fit Index (NFI) = 0.932, Root Mean Square Residual (RMR) = 0.079, Goodness of Fit Index (GFI) = 0.895, Incremental Fit Index (IFI) = 0.959, Relative Fit Index (RFI) = 0.921, Root-Mean Square Error of Approximation (RMSEA) = 0.05.

***p < 0.001.

In the tourists’ satisfaction context, the investigated respondents perceived a higher level of overall satisfaction where the average mean for all attributes ranged from 4.01 to 4.08. The statement **”**compared with other retreats, the level of satisfaction in this place was amazing**”** was perceived as the highest one. Furthermore, they ranked **”**I will certainly recommend this retreat/studio to my friends and acquaintances**”** with an average mean of 4.24 as the highest item associated with tourists’ behavioral intentions.

### Measurement model

As mentioned previously the study data was gathered by using a self-administrated questionnaire. Consequently, a CMV was firstly determined by using Harman’s single-factor test. As a result, one component was found to account for only 37% (less than 50%) of the variance which reveals that method bias/variance does not represent a problem (Podsakoff et al., 2003).

To explore the validity and reliability of the study constructs, CFA using maximum Likelihood was undertaken. As shown in Table 5, values of Cronbach’s alpha and composite reliability (CR) of all latent constructs were over the 0.7 thresholds suggested by Hair et al. (2013), specifying acceptable internal reliability. Convergent and discriminant validity were used to examine the construct validity (Chin et al., 1997). Convergent validity requires all standardized item loadings to be more than the threshold (>0.50) and the average variance extracted (AVE) exceeds 0.50 as recommended by Duckworth and Kern (2011) and Hair et al. (2013). The factor loading for all study items is higher than 0.50 and the AVE of each construct was above 0.50, ranging from 0.552 to 0.775, which confirms the adequacy of convergent validity of study constructs. Based on the criterion of Fornell–Larcker, the constructs’ discriminant validity requires the square root of AVE of every construct to be higher than its correlation with other constructs. As shown in Table 6, results indicated that the AVE square root of all constructs is higher than their correlations with other ones which means that discriminant validity has been achieved (Hair et al., 2013).

**TABLE 6:** Discriminant validity based on Fornell–Larcker criterion.

Construct	1	2	3
(1) Perceived service quality	**0.852**		
(2) Tourist satisfaction	0.303	**0.853**	
(3) Tourists’ behavioral intentions	0.738	0.616	**0.743**

Bold diagonal numbers represent the square root of AVE’s study constructs.

The goodness of fit indices of the study model were good; *x*^2^ = 486.312 (df = 201) *p* < 0.001, x^2^/df = 2.419, Comparative Fit Index (CFI) = 0.958, Normed Fit Index (NFI) = 0.932, Root Mean Square Residual (RMR) = 0.079, Goodness of Fit Index (GFI) = 0.895, Incremental Fit Index (IFI) = 0.959, Relative Fit Index (RFI) = 0.921, Root-Mean Square Error of Approximation (RMSEA) = 0.05.

### Structural equation modeling

In order to identify the direction as well as the relationship between the study’s constructs, SEM has been utilized. According to the goodness of fit indices, the study’s model fit was acceptable (see Table 6).

**FIGURE 2:**
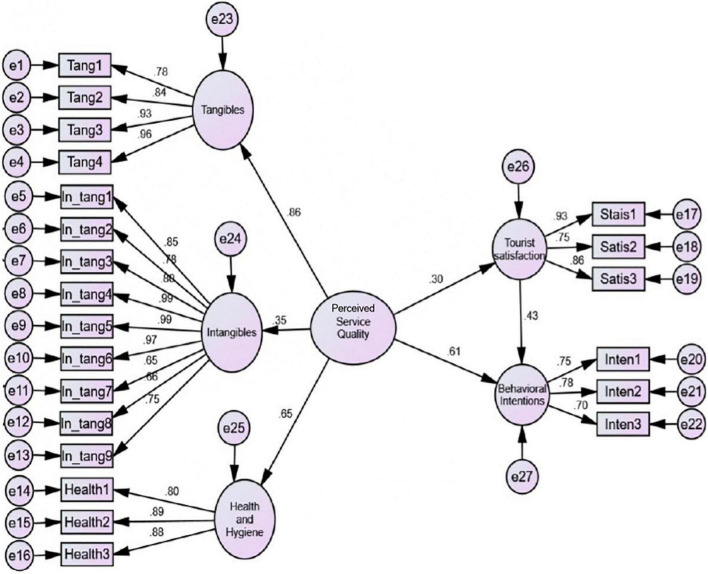
The structural model.

Regarding the relationship between the study dimensions, the results in Table 7 indicated a positive relationship between all study dimensions. PSQ is highly correlated with tourists’ behavioral intentions and customer satisfaction respectively. In addition, the findings of SEM, presented in Table 8 and Figure 2, illustrated that PSQ impacts positively and significantly on tourists’ satisfaction (β = 0.303, *t*-value = 4.299, *P* < 0.001). As a result, hypothesis 1 is supported. Furthermore, PSQ has a high positive and significant effect on tourists’ behavioral intention (β = 0.608, *t*-value = 7.199, *P* < 0.001), and accordingly, H2 is accepted. About the direct relationship between tourist satisfaction and tourists’ behavioral intentions, the findings of SEM support H3 which predicted that yoga tourist satisfaction significantly and positively affects tourists’ behavioral intentions (β = 0.431, *t*-value = 7.434, *P* < 0.001). To examine the mediation effect of tourist satisfaction in the relationship between PSQ and tourists’ behavioral intentions, the path was reviewed using the suggestions of Kelloway (1995) and Zhao et al. (2010) for partial and full mediation. They illustrated that only full mediation can be established if the indirect effects are significant, while the direct effects are not; partial mediation occurs when both paths are significant. Consequently, the findings of the SEM revealed that tourists’ satisfaction partially and significantly mediates the positive relationship between PSQ and yoga tourists’ behavioral intentions, where the two paths (direct and indirect) are significant. Upon that H4 is accepted.

**TABLE 7:** Correlation matrix for the investigated dimensions.

	Tangibles	Intangibles	Health and hygiene	Perceived service quality	Tourist satisfaction	Tourists’ behavioral intentions
Tangibles	1					
Intangibles	0.299	1				
Health and Hygiene	0.518	0.208	1			
Perceived service quality	0.628	0.911	0.509	1		
Tourist satisfaction	0.218	0.221	0.328	0.363	1	
Tourists’ behavioral intentions	0.261	0.413	0.562	0.649	0.527	1

**TABLE 8:** Structural parameter estimates.

Hypothesized path				Standardized path coefficients	*t*-value	Results
**H_1_**: Perceived service quality	→	Tourist satisfaction		0.303***	4.299	Supported
**H_2_**: Perceived service quality	→	Tourists’ behavioral intentions		0.608***	7.199	Supported
**H_3_**: Tourist satisfaction	→	Tourists’ behavioral intentions		0.431***	7.434	Supported
**H_4_**: Perceived service quality →	Tourist satisfaction	→	Tourists’ behavioral intentions	0.131*	3.109	Supported

***P < 0.001, *P < 0.50.

## Discussion and implications

### Discussion

The main objective of this research study was to investigate the impact of PSQ on tourist satisfaction and behavioral intentions and explore the potential mediating role of tourist satisfaction in the relationship between service quality and behavioral intentions in the yoga tourism context during the COVID-19 pandemic, in a sample of Egyptian tourist destinations, particularly, South Sinai governorate. Upon the previous literature, the conceptual model proposed in this study hypothesized that PSQ affects significantly and positively yoga tourists’ behavioral intentions both directly and indirectly through tourist satisfaction. Furthermore, the model also postulated that PSQ has a significant and positive impact on yoga tourist satisfaction, and yoga tourist satisfaction also has a significant and positive impact on yoga tourists’ behavioral intentions. Accordingly, the following findings will be discussed in accordance with the literature reviewed.

Regarding the profile of yoga tourists, the findings of this study are following the findings of the previous studies that indicated that yoga tourists predominantly are single females, with middle age ranging from 30 to 45 years, and had a higher level of education (Lehto et al., 2006; Kelly and Smith, 2009; Ali-Knight and Ensor, 2017).

In response to the COVID-19 pandemic, tourists have become more attentive to the quality of public health services during their travel (Han et al., 2021). The results of this study indicated that the investigated yoga tourists perceived enhancing physical conditions and mental well-being as the vital factors that affect their choice of yoga trip in the era of COVID-19. These results are in line with the findings of the previous study which confirmed that yoga as an ancient science, could be able to promote health for the body, peace for the mind, joy for the heart, and liberation for the soul (Bowers and Cheer, 2017). These findings also support the results of Lehto et al. (2006) who concluded that relaxing, renewing yourself, and being more flexible in body and mind were the most motivations for going on yoga trips. In contemporary societies, yoga has gained popularity as a powerful solution for reducing stress and pressures that are exerted on individuals (Shinn and Viron, 2020). Furthermore, the findings of this study revealed that the investigated participants perceived health and hygiene regulations (COVID-19 preventative measures) as the most important factor that affects their choice of yoga trips. This finding is inconsistent with the previous study which concluded that good and experienced teachers are the most predictor of yoga trip choice (Ali-Knight and Ensor, 2017). Researchers believe that the reason for this result may refer to the increasing health awareness among tourists and their keenness to follow up the precautionary measures to prevent COVID-19 from spreading.

The study findings revealed that the investigated respondents highly PSQ specifically health and hygiene quality attributes. The yoga service providers were keen to adopt preventative measures to eliminate the spreading of COVID-19. They keep rooms, bathrooms, and public areas usually cleaned and well sanitized. Protective masks and hand sanitizers were available for all guests and staff, and space and seating capacity were limited to allow for social distancing. These findings are consistent with the regulations of World Health Organization [WHO] (2021). Moreover, to foster the yogis’ experience, yoga service providers offered comfortable furniture, fixture, and an appropriate atmosphere, and the service staff was keen to appear well-groomed and in good personal hygiene. Staff also was committed to showing a sincere interest in solving guests’ complaints, providing services at the time they promise to do so, and telling exactly the right schedule of the services to be performed. Consequently, investigated participants were highly satisfied with their experience and highly intent to recommend the retreats to their friends and relatives, communicate positive WoM to their peers and acquaintances, and revisit the place in the future, if they had a chance. These findings are consistent with various previous studies (i.e, González et al., 2007; Ladhari, 2009; Saha and Theingi., 2009; Zeng and Yi Man Li, 2021) which confirmed that a higher level of PSQ improves tourists’ satisfaction and fosters their behavioral intentions.

In the context of the interrelationship between the study’s constructs (PSQ, tourist satisfaction, and tourists’ behavioral intentions), it could be concluded that; the results of the current study are in accordance with the findings of previous studies (i.e., Zeithaml et al., 1996; Baker and Crompton, 2000; Bei and Chiao, 2001; Alexandris et al., 2002; Ladhari, 2009) which confirmed that the higher PSQ affects directly, positively, and significantly behavioral intentions. The findings of the current study suggest that yoga tourists will likely establish a positive attitude and future intention if they perceived a higher level of service quality. Consequently, they will be encouraged to recommend the retreat and destination which they have visited their friends and relatives, and they will return to the same yoga service providers in the future.

In the tourism context, the findings of the study are consistent with the findings of an empirical study conducted by Lee et al. (2004) which revealed that service quality has a positive and direct significant impact on the behavioral loyalty of forest visitors. Further, these findings support the results of Cham et al. (2014), in the context of medical tourism, which suggested that PSQ had a positive, direct, and significant impact on tourists’ behavioral intentions (i.e., intention to revisit). Similar findings have been illustrated by González et al. (2007) who empirically concluded that PSQ significantly and positively affects tourists’ WoM communication, buying intentions, and price sensitivity.

The findings of the study also found that PSQ has a positive and significant impact on tourist satisfaction. This finding supports the previous findings in different contexts such as health and wellness tourism (Quintela et al., 2010), hotel industry (Ladhari, 2009), rural tourism (Osman and Sentosa, 2013), low-cost airline carriers (Saha and Theingi., 2009; Hassan and Salem, 2021) which assured that PSQ is a key antecedent of tourist satisfaction. As a result, a higher PSQ fosters yoga tourists’ satisfaction. These findings are in accordance with that revealed by Quintela et al. (2010) who investigate the relationship between PSQ and tourist satisfaction among Portuguese tourists and indicated that PSQ had a significant positive impact on tourist satisfaction. Furthermore, in the Edu-tourism context, Edu-tourist satisfaction was positively and significantly influenced by university- PSQ (Rahimizhian et al., 2020). In the context of cultural heritage tourism, these findings also foster Canny’s study findings (Canny, 2012) which revealed local tourists’ satisfaction is significantly impacted by PSQ.

Regarding the relationship between tourist satisfaction and tourists’ behavioral intentions, the study findings confirmed the significant positive relationship between them. This finding is consistent with other results which confirmed that tourist satisfaction plays a vital role in increasing tourists’ behavioral intentions in engaging in WoM activities, recommending to others, and revisiting intentions (Tian-Cole et al., 2002; Cole and Illum, 2006). These findings are in line with that concluded by Viana et al. (2021) who concluded a significant positive relationship between tourist satisfaction and behavioral intentions among 400 tourists who visited Ramelau Mountain. Moreover, these results support the findings of Chen and Chen (2010) who revealed that the more satisfied tourists are with experiences, the more likely they are to revisit and recommend the destination to others. In addition, the findings of the current study reinforce the results of Hutchinson et al. (2009), who examined the relationship between satisfaction and behavioral intentions among golf travelers and concluded that tourist satisfaction is a key predictor of behavioral intentions.

Furthermore, the findings of the study confirmed the indirect significant relationship (through tourist satisfaction) between PSQ and tourists’ behavioral intentions. The findings of the current study illustrated that the direct effect of PSQ on behavioral intention (0.608) was higher than the indirect impact (0.131). Consequently, tourist satisfaction partially mediates the relationship between constructs. These findings are matching with that concluded by Cham et al. (2014) who revealed that satisfaction plays a partial mediating role in the relationship between PSQ and behavioral intentions among medical tourists. Further, Osman and Sentosa (2013) confirmed that tourist satisfaction has a significant and positive partial mediating effect on the association between service quality and tourists’ loyalty in the rural tourism context. Bei and Chiao (2001) concluded that PSQ positively, significantly, and indirectly (through satisfaction) affects consumers’ loyalty. This finding also supports the finding of Reyes Vélez et al. (2019) who found that satisfaction partially mediated the association between perceived tour service quality and tourists’ behavioral intentions in an island context. On the other hand, this finding is inconsistent with that concluded by Cole and Illum (2006) who showed that tourist satisfaction fully mediates the relationship between PSQ and behavioral intentions among rural heritage festival visitors.

### Theoretical implications

The study has some implications for scholars, especially tourism ones. Firstly, the study would add a significant contribution to fostering the general body of hypothetical literature related to service quality, tourist satisfaction, and tourists’ behavioral intentions interrelationship in the yoga tourism context. The study offers evidence for the direct, as well as indirect effects of PSQ (through tourist satisfaction) on yoga tourists’ behavioral intentions. Secondly, to the best of our knowledge, this might be the first empirical study that explores, directly and indirectly, the attitudinal and behavioral consequences of PSQ in the yoga tourism context in the era of COVID-19 specifically in one of the developing countries contexts (i.e., Egypt). Thirdly, the study developed a valid service quality scale including a new dimension **”**Health and Hygiene**”** besides tangibles and intangibles ones that would help scholars in their further research that examine PSQ during health epidemics such as COVID-19, in particular. Fourthly, the findings of the study could help tourism scholars in exploring the profile of yoga tourists, their motivations, factors influencing their choice of yoga trips as well as service quality dimensions that affect future behavioral intention and satisfaction in the tourism industry specifically in crisis times (i.e., COVID-19 pandemic), which considered a basis to further studies aim to examine justifications for improving tourists’ satisfaction and enhancing their behavioral intentions in the yoga tourism context. Fifthly, the current study suggests that satisfaction and behavioral intentions to revisit, willingness to recommend to others, and engaging in positive WoM communication as consequences of PSQ. Moreover, satisfaction is a key predictor of behavioral intentions. Hence, sufficient consideration must be paid to these variables for ensuring yoga tourists’ behavioral intentions.

### Practical implications

Upon the study findings, several implications for yoga service providers and tourism professionals specifically in the developing countries context should be considered. From a practical point of view, the findings of the current study provide valuable guidelines for destination marketers in the context of yoga tourism destinations. As a first priority, destination marketers should strive to improve the quality of tourism and hospitality services provided. Based on our findings, PSQ is positively associated with tourist satisfaction and behavioral intentions. In order to meet the needs and desires of yoga tourists, destination marketers should take strenuous measures to improve the three latent dimensions of service quality (tangibles, intangibles, and health and hygiene). The higher level of PSQ observed in the investigated yoga retreats/studios seems clear. Although these levels have increased yoga tourists’ behavioral intentions, more efforts should be considered to determine and involve the factors that improve tourist satisfaction which play a significant role in this relationship. In particular, yoga service providers should be aware that, among the various dimensions of service quality, health and hygiene are highly correlated to tourists’ satisfaction and behavioral intentions. As a result, responses to COVID-19, considering health and hygiene regulations, as well as adopting COVID-19 precautionary measures in yoga retreats are becoming significantly important.

It is apparent that intangibles play a critical impact in both tourist satisfaction and behavioral intentions. As a result, improving yoga tourists’ behavioral intentions and satisfaction with their travel experience requires yoga service providers to take care of giving tourists personalized attention, understanding, fulfilling their specific needs and properly responding to their requests. Professional competence of yoga teachers, healthy food and safety, comfortable accommodation services and facilities, as well as recreational activities and cultural attractions are vital approaches for yoga tourism marketing that should be considered by yoga tour operators. Yoga styles that promote physical and mental well-being should be considered when designing a yoga excursion package. Measuring customer satisfaction periodically is essential. Tourists’ feedback, recommendations, and comments should be considered to improve the yogis’ behavioral intentions.

## Limitations of the study and further research

The present study has some limitations as follows; the first one is related to the generalizability of the study findings. The subject of this study was yoga tourists staying in yoga retreats/studios in Egyptian destinations (South Sinai Governorate). Generalizations of the study findings would be difficult. Further research may focus on other geographical areas. The second limitation pertains to the method of data collection, where the study utilized a self-administrated questionnaire where the investigated participants answer according to their subjective perspectives. A mixed-method approach (quantitative and qualitative) may provide a better understanding. The third is concerned with the limitation of behavioral intentions’ variables, where the study only examined three variables that related to revisiting intention, recommendation, and positive WoM. Further studies may include other variables such as intention to visit, tourists’ loyalty, and the probability of paying more. Further research may focus on other influential variables of yoga tourists’ satisfaction and behavioral intentions such as perceived value, destination image as well as tourists’ expectations. Investigating the role of yoga tourism in enhancing the quality of life in the era of COVID-19 is a vital issue. Strategical marketing plans for yoga tourism should be considered.

## Data availability statement

The original contributions presented in this study are included in the article/supplementary material, further inquiries can be directed to the corresponding author.

## Ethics statement

The studies involving human participants were reviewed and approved by the deanship of the scientific research ethical committee, King Faisal University. The patients/participants provided their written informed consent to participate in this study.

## Author contributions

AHA and SM: conceptualization, data curation, and supervision. AHA and AK: methodology. AHA and AIA: software. AHA, AK, and SM: validation, resources, and funding acquisition. AHA, AJA, and SM: formal analysis and writing—review and editing. AHA, AJA, and AIA: investigation. AHA, AIA, and AK: writing—original draft preparation. AHA, SM, and AK: visualization. AHA: project administration. All authors have read and agreed to the published version of the manuscript.
